# Pre-Treatment with Allopurinol or Uricase Attenuates Barrier Dysfunction but Not Inflammation during Murine Ventilator-Induced Lung Injury

**DOI:** 10.1371/journal.pone.0050559

**Published:** 2012-11-30

**Authors:** Maria T. Kuipers, Hamid Aslami, Alexander P. J. Vlaar, Nicole P. Juffermans, Anita M. Tuip-de Boer, Maria A. Hegeman, Geartsje Jongsma, Joris J. T. H. Roelofs, Tom van der Poll, Marcus J. Schultz, Catharina W. Wieland

**Affiliations:** 1 Laboratory of Experimental Intensive Care and Anesthesiology, Academic Medical Center, University of Amsterdam, Amsterdam, The Netherlands; 2 Center of Experimental and Molecular Medicine, Academic Medical Center, University of Amsterdam, Amsterdam, The Netherlands; 3 Division of Infectious Diseases, Academic Medical Center, University of Amsterdam, Amsterdam, The Netherlands; 4 Department of Intensive Care, Academic Medical Center, University of Amsterdam, Amsterdam, The Netherlands; 5 Department of Pathology, Academic Medical Center, University of Amsterdam, Amsterdam, The Netherlands; French National Centre for Scientific Research, France

## Abstract

**Introduction:**

Uric acid released from injured tissue is considered a major endogenous danger signal and local instillation of uric acid crystals induces acute lung inflammation via activation of the NLRP3 inflammasome. Ventilator-induced lung injury (VILI) is mediated by the NLRP3 inflammasome and increased uric acid levels in lung lavage fluid are reported. We studied levels in human lung injury and the contribution of uric acid in experimental VILI.

**Methods:**

Uric acid levels in lung lavage fluid of patients with acute lung injury (ALI) were determined. In a different cohort of cardiac surgery patients, uric acid levels were correlated with pulmonary leakage index. In a mouse model of VILI the effect of allopurinol (inhibits uric acid synthesis) and uricase (degrades uric acid) pre-treatment on neutrophil influx, up-regulation of adhesion molecules, pulmonary and systemic cytokine levels, lung pathology, and regulation of receptors involved in the recognition of uric acid was studied. In addition, total protein and immunoglobulin M in lung lavage fluid and pulmonary wet/dry ratios were measured as markers of alveolar barrier dysfunction.

**Results:**

Uric acid levels increased in ALI patients. In cardiac surgery patients, elevated levels correlated significantly with the pulmonary leakage index. Allopurinol or uricase treatment did not reduce ventilator-induced inflammation, IκB-α degradation, or up-regulation of NLRP3, Toll-like receptor 2, and Toll-like receptor 4 gene expression in mice. Alveolar barrier dysfunction was attenuated which was most pronounced in mice pre-treated with allopurinol: both treatment strategies reduced wet/dry ratio, allopurinol also lowered total protein and immunoglobulin M levels.

**Conclusions:**

Local uric acid levels increase in patients with ALI. In mice, allopurinol and uricase attenuate ventilator-induced alveolar barrier dysfunction.

## Introduction

The acute respiratory distress syndrome (ARDS) is a devastating pulmonary condition characterized by acute inflammation, loss of alveolar-capillary membrane function, and neutrophilic alveolitis [1], [2]. Mechanical ventilation (MV), often mandatory in the management of these patients, can enhance lung injury by repetitive opening, collapsing, and overstretching of alveoli, termed ventilator-induced lung injury (VILI) [3]. The ARDS network demonstrated that protective ventilator settings improve outcome in ARDS patients [4]. However, to date reported mortality rates remain up to 42% demanding the search for additive therapeutic strategies [5].

Bacterial products activate pattern recognition receptors such as the Toll-like receptors (TLRs) and Nod-like receptors (NLRs) triggering an inflammatory response necessary to combat infections [6]. In the last decade it became clear that injured tissue releases endogenous danger molecules, referred to as damage-associated molecular patterns (DAMPs) or alarmins, which trigger the same immune receptors and initiate inflammation in the absence of infection [6]. Hyperinflammation and immune dysregulation are characteristic for VILI. Animal studies demonstrated up-regulation of TLR4 by MV [7], [8] and indicated that MV-induced inflammation is TLR4 dependent [7], [9]. In a more recent study we showed that VILI is also mediated by NLRP3 inflammasome activation [10]. Since these experiments were performed in uninfected animals it is thought that pattern recognition receptors are activated by exposure to endogenous danger molecules. Studies indeed demonstrated that DAMPs are released as a consequence of (injurious) MV [11].

Uric acid, the product of purine catabolism, is considered a major DAMP contributing to cell death-induced acute inflammation [12]. The relevance of extracellular uric acid in lung pathology was previously demonstrated in a bleomycin-induced lung injury model in mice [13]. Local administration of uric acid crystals induced acute lung inflammation, a process depending on the NLRP3 inflammasome, the combined action of TLR2 and TLR4, the interleukin (IL)-1 receptor and myeloid differentiation factor 88 pathways. Treatment with allopurinol (blocking uric acid synthesis) greatly inhibited bleomycin-induced inflammation and resulted in reduced neutrophil influx and lower levels of keratinocyte-derived chemokine (KC) and IL-1β in the lung [13]. Uricase treatment (rapidly degrading uric acid) also reduced neutrophil recruitment and pulmonary IL-1β production in these animals. Recently, we demonstrated that uric acid levels in bronchoalveolar lavage fluid (BALF) increase upon injurious MV in mice [10]. These findings suggest that uric acid might also contribute as a pro-inflammatory DAMP in VILI. Allopurinol and uricase treatment are already well-established therapies to lower pathogenic uric acid concentrations in patients suffering from gout or to prevent tumor lysis syndrome during chemotherapy. In the present study we hypothesized that allopurinol and uricase attenuate pulmonary inflammation and barrier dysfunction compared to vehicle pre-treated mice in a clinically relevant model of VILI. In addition, we hypothesized that BALF uric acid levels are also increased in patients with lung injury. To study this, we measured BALF uric acid levels in patients suffering from acute lung injury (ALI). In a different patient cohort BALF uric acid levels were correlated with pulmonary leakage index (PLI) a measure of microvascular pulmonary permeability.

**Table 1:** Primers used for real-time RT-PCR.

	Forward		Reverse	
**NLRP3**	5′-ccacagtgtaacttgcagaagc-3′		5′-ggtgtgtgaagttctggttgg-3′	
**TLR2**		5′-ggggcttcacttctctgctt-3′		5′-agcatcctctgagatttgacg-3′
**TLR4**		5′-ggactctgatcatggcactg-3′		5′-ctgatccatgcattggtaggt-3′
**ICAM-1**		5′-ggagacgcagaggaccttaacag-3′		5′-cgacgccgctcagaagaacc-3′
**VCAM**		5′-tgaagttggctcacaattaagaagtt-3′		5′-tgcgcagtagagtgcaagga-3′
**E-selectin**		5′-caacgtctaggttcaaaacaatcag-3′		5′-ttaagcaggcaagaggaacca-3′
**HPRT**		5′-tcctcctcagaccgctttt-3′		5′-cctggttcatcatcgctaatc-3′

NLRP3 =  Nod-like receptor 3, TLR = Toll-like receptor, ICAM = intracellular adhesion molecule;

VCAM = vascular cell adhesion molecule; HPRT = hypoxanthine-guanine phosphoribosyl transferase.

## Materials and Methods

### Patients

The Medical Ethics Committee from the University of Amsterdam approved both study protocols.

**Figure 1:**
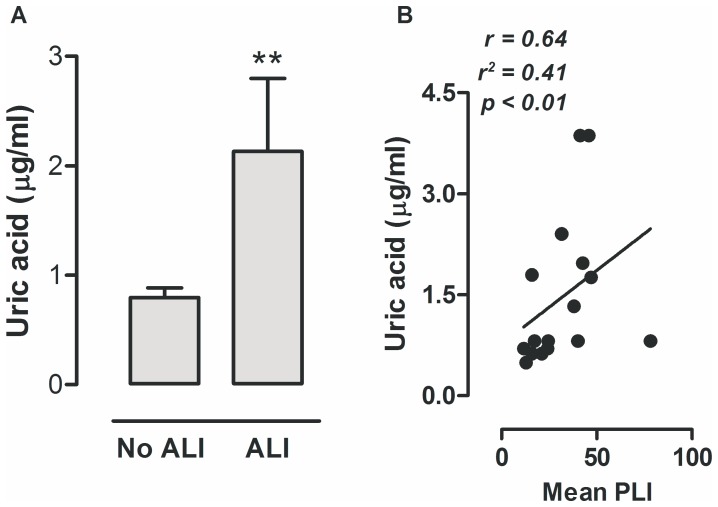
Uric acid levels in ventilated patients. Levels of uric acid in bronchoalveolar lavage fluid obtained from patients with acute respiratory distress syndrome (ARDS) (n = 16) and patients without ARDS (n = 62) (**A**). In a different cohort of cardiac surgery patients elevated uric acid levels in lung lavage fluid were correlated with the mean pulmonary leakage index (PLI) measured, displayed in ×10^−3^ min^−1^ (**B**) (n = 16). Data are presented as mean ± SEM. **p<0.01 vs no ALI.

Uric acid measurements were performed on samples obtained during previous studies where the relation between transfusion and the onset of ALI in adult cardiac surgery patients was investigated [14], [15]. Written informed consent for participation in the study was asked and obtained prior to surgery. Exclusion criteria were emergency surgery and pulmonary thrombo-endarterectomy. A detailed description of study design and methods was described previously and is also provided in Data S1 [14], [15].

**Figure 2:**
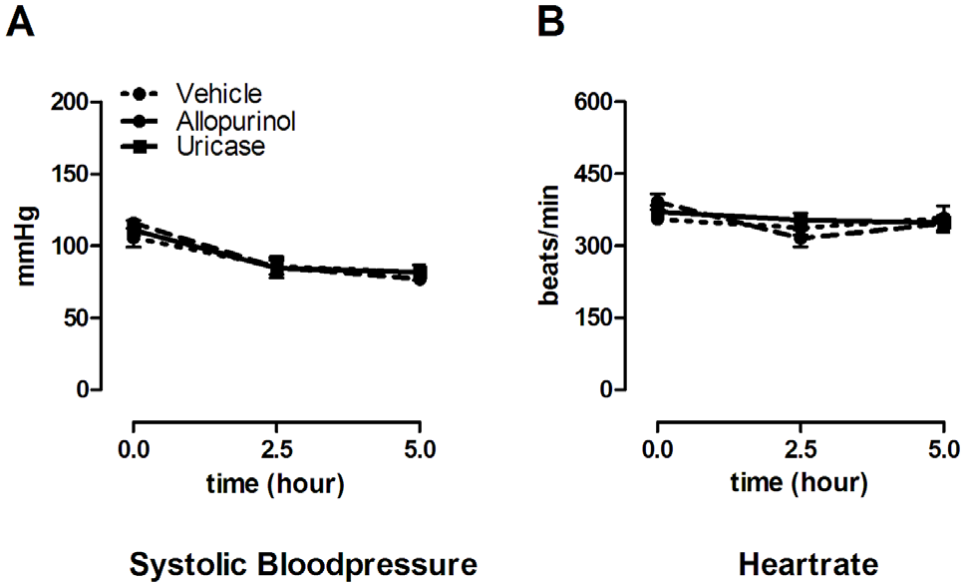
Hemodynamic parameters during 5 hours of ventilation in mice. Hemodynamic parameters of mice pre-treated with vehicle (10% dimethylsulfoxide), uricase (0.2 mg/kg), or allopurinol (25 mg/kg) 1 hour before start of mechanical ventilation. Systolic bloodpressure (**A**) and heart rate (**B**) was measured at start of ventilation, after 2.5, and after 5 hours. Data represent mean ± SEM of n = 9 mice per group.

**Figure 3:**
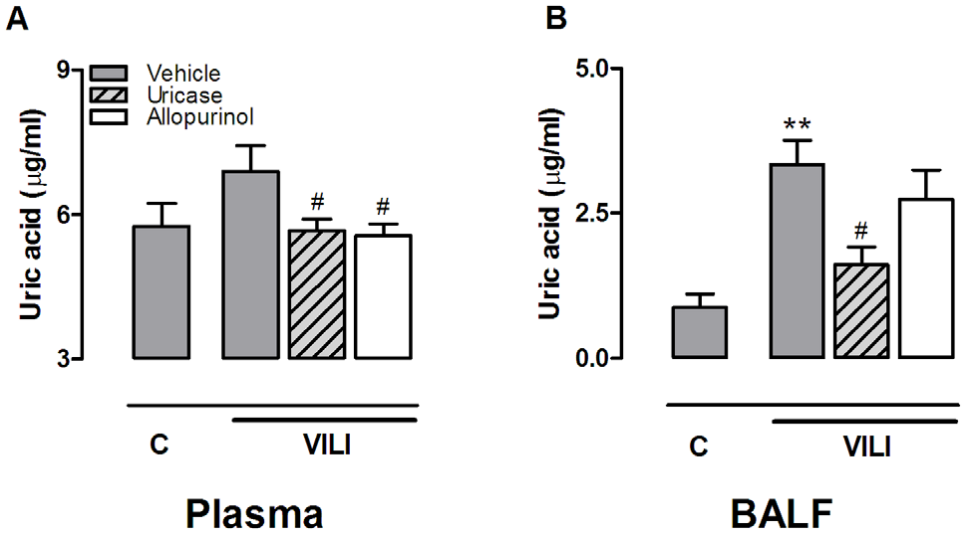
Uric acid levels in murine ventilator-induced lung injury. Uric acid levels in plasma (**A**) and bronchoalveolar lavage fluid (BALF) (**B**) of mice pre-treated with vehicle (10% dimethylsulfoxide, dark grey bars), uricase (0.2 mg/kg, light grey, striped bars), or allopurinol (25 mg/kg (white bars) 1 hour before start of 5 hours of mechanical ventilation (VILI). Spontaneously breathing, vehicle pre-treated mice served as controls (C). Data represent mean ± SEM of 4 control mice and n = 6−9 ventilated mice. **p<0.01 vs. control, #p<0.05 vs. vehicle ventilated.

**Figure 4:**
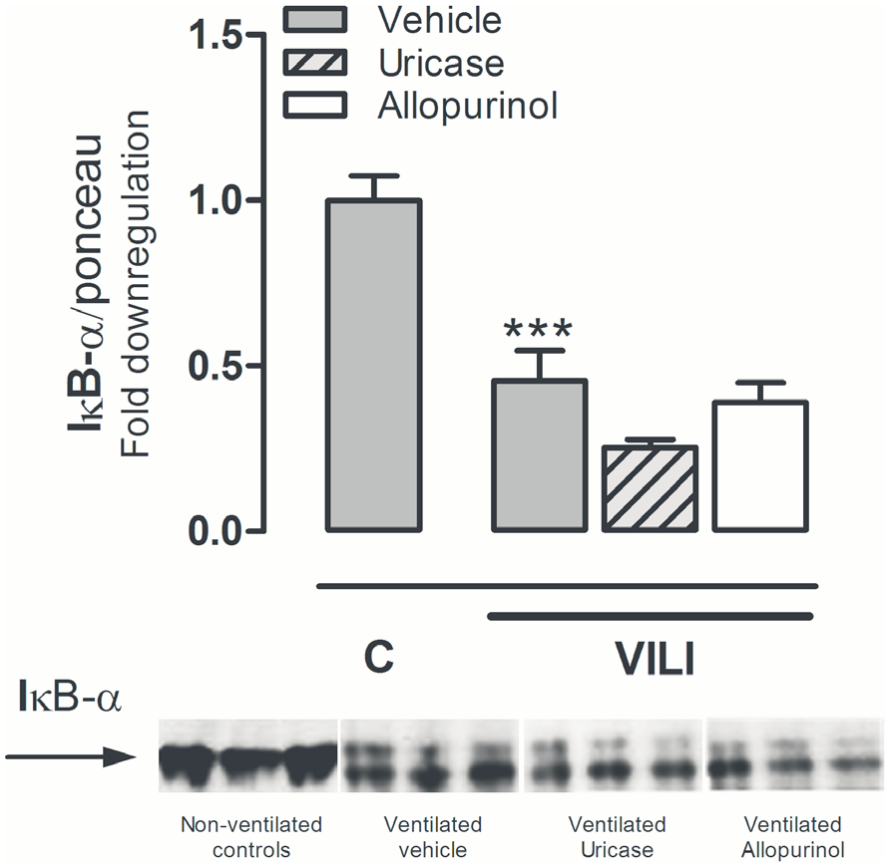
Pulmonary inhibitory kappa-B alpha levels in murine ventilator-induced lung injury. Fold down regulation of inhibitory kappa-B alpha (IκB-α) in lung tissue homogenate relative to total protein levels measured by Ponceau staining. Mice were pre-treated with vehicle (10% dimethylsulfoxide), uricase (0.2 mg/kg, light grey, striped bars), or allopurinol (25 mg/kg (white bars) 1 hour before start of mechanical ventilation (VILI). Spontaneously breathing, vehicle pre-treated mice served as controls (C). After 5 hours mice were killed. Data represent mean (SEM) of 4 control mice and n = 5−6 ventilated mice. ***p<0.001 vs. vehicle control.

**Table 2:** Pulmonary mRNA levels of pattern-recognition receptors.

		C	MV					
		Vehicle	Vehicle		Uricase		Allopurinol	
**TLR2/HPRT**	0.12 [0.12]		10.7 [1.68]**	12.3 [1.91]		10.5 [2.16]		
**TLR4/HPRT**	2.39 [0.76]		9.21 [1.06]**	8.02 [1.01]		8.78 [0.98]		
**NLRP3/HPRT**	0.93[0.17]		2.23 [0.20]	3.38 [0.54]		2.82 [0.44]		

Animals were pre-treated with uricase (0.2 mg/kg), allopurinol (25 mg/kg), or vehicle (10% dimethylsulfoxide) and were mechanically ventilated for 5 hours (MV), non-ventilated vehicle pre-treated mice served as controls (C). Gene expression levels of Toll-like receptor (TLR) 2, TLR4, and, NLRP3 were measured in lung tissue homogenates and normalized to the house-keeping gene hypoxanthine-guaninephosphoribosyl transferase (HPRT). Data represent mean ± SEM of n = 4 non-ventilated mice and n = 8−9 ventilated mice/group.

** p<0.01 versus non-ventilated controls.

#### Uric acid measurements in human lung lavage fluid

Patients were observed for the onset of ALI up to 30 hours after cardiac surgery [14]. At onset of ALI, a non-directed bronchoalveolar lavage was performed as described before [16]. Controls were patients not developing ALI and lavaged within 30 hours of ICU admission. In total, 16 transfusion-related ALI and 62 control cases were analyzed in this study.

**Figure 5:**
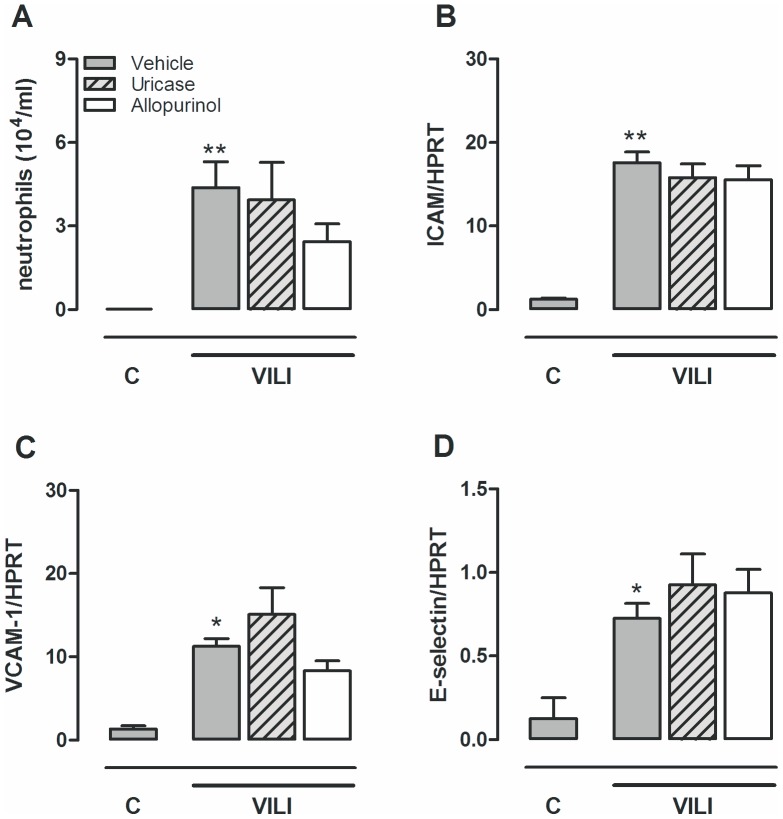
Allopurinol or uricase pre-treatment do not reduce neutrophil influx. Mice were pre-treated with vehicle (10% dimethylsulfoxide, dark grey bars), uricase (0.2 mg/kg, light grey, striped bars), or allopurinol (25 mg/kg (white bars) 1 hour before start of mechanical ventilation (VILI). Spontaneously breathing, vehicle pre-treated mice served as controls (C). After 5 hours mice were killed. Neutrophil influx into the alveolar compartment (**A**) and the pulmonary expression of intracellular adhesion molecule-1 (ICAM-1) (**B**), vascular adhesion molecule (VCAM) (**C**) and e-selectin (**D**) relative to the housekeeping gene HPRT were analyzed. Data represent mean ± SEM of 4 control mice and n = 7−9 ventilated mice. *p<0.05, **p<0.01 vs. vehicle control.

**Figure 6:**
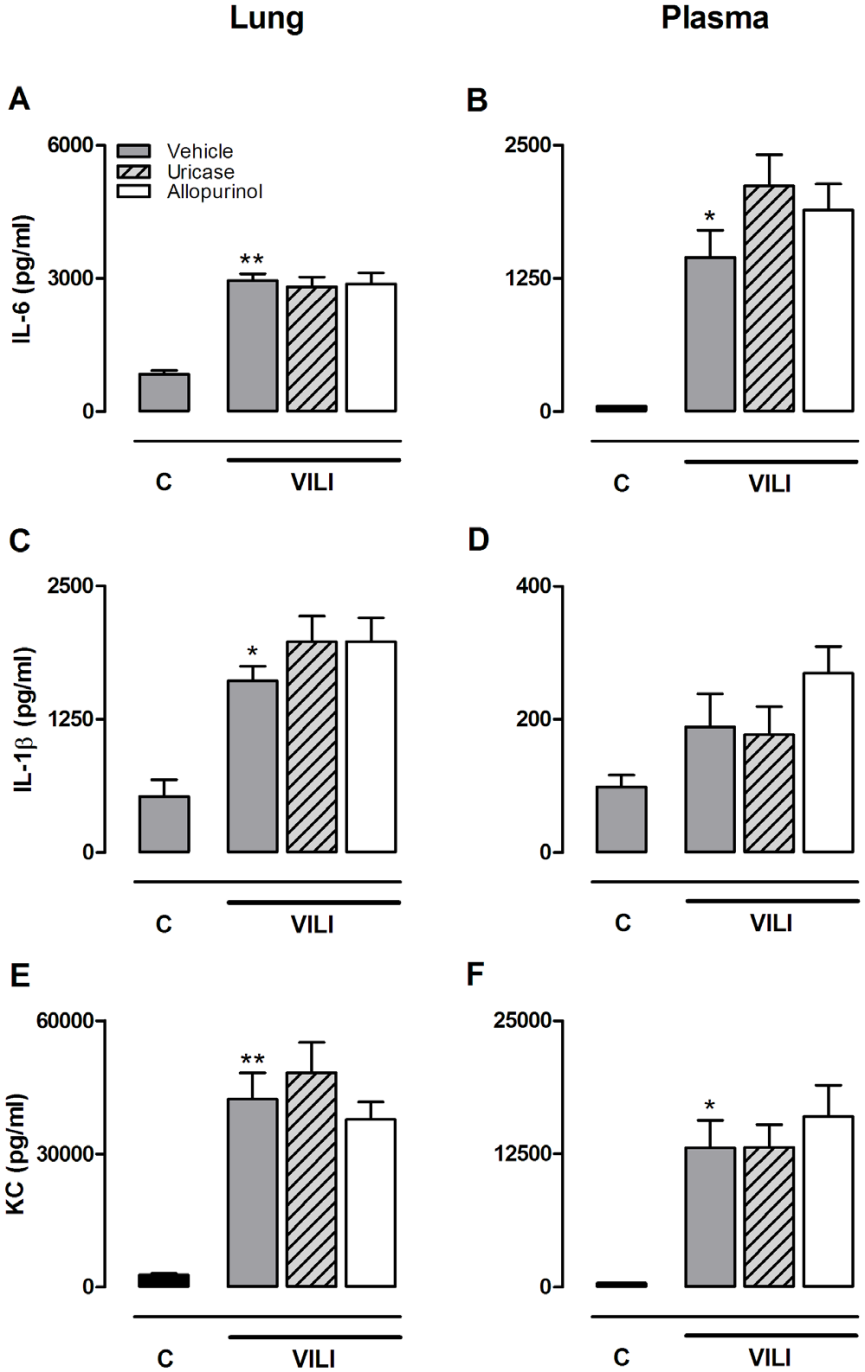
Allopurinol and uricase pre-treatment do not affect pulmonary and systemic cytokine levels. Mice were pre-treated with vehicle (10% dimethylsulfoxide, dark grey bars), uricase (0.2 mg/kg, light grey, striped bars), or allopurinol (25 mg/kg (white bars) 1 hour before start of mechanical ventilation (VILI). Spontaneously breathing, vehicle pre-treated mice served as controls (C). After 5 hours mice were killed. Interleukin (IL)-6 (**A,B**), IL-1β (**C,D**) and keratinocyt-derived chemokine (KC) (**E,F**) levels were determined in lung tissue homogenate and plasma. Data represent mean ± SEM of 4 control mice and n = 9 mice per ventilated group. *p<0.05, **p<0.01 vs. vehicle control.

#### Association between uric acid levels and pulmonary leakage index

Pulmonary leakage index (PLI) can be used as a measure of microvascular pulmonary permeability and has been shown to be an early marker of acute lung injury in at risk patients [17]. In a separate cohort of 60 cardiac surgery patients, pulmonary leakage index (PLI) was measured [15]. Transferrin was labelled *in vivo*, after intravenous injection of ^67^Gallium (Ga)-citrate, 4.5 MBq (physical half-life 78 hour; Mallinckrodt Diagnostica, Petten, The Netherlands). In supine position, two scintillation detection probes (Eurorad C.T.T., Strasburg, France) were positioned over the right and left lung apices of the patient. When ^67^Ga was injected, radioactivity was measured during 30 minutes. The ^67^Ga counts were corrected for background radioactivity, physical half-life, spillover of ^67^Ga, obtained by *in vitro* measurement of ^67^Ga, and expressed as cpm per lung field. Blood samples were taken at 0, 5, 8, 12, 16, 20, 25 and 30 min after ^67^Ga injection. Each sample was weighed and radioactivity was determined using a single-well well-counter (LKB Wallac 1480 WIZARD, Perkin Elmer, Life Science, Zaventem, Belgium), background, spillover of ^67^Ga, and decay were taken into account. Results are expressed as cpm g^–1^. For each blood sample, a time-matched count rate over each lung was defined. The radioactivity ratio was calculated as (^67^Ga_lung_)/(^67^Ga_blood_) and plotted against time. The PLI was calculated from the slope of increase of the radioactivity ratio divided by the intercept, physical factors in radioactivity detection were corrected this way. The PLI represents the transport rate of ^67^Ga-transferrin from the intravascular to the extravascular lung space and is therefore a measure of pulmonary vascular permeability. The values for both lung fields are averaged. Measurement error is ∼10% and the upper limit of normal for PLI is 14.7×10^–3^/min. The present analysis included the patients with a PLI measurement and from whom also bronchoalveolar lavage fluid was obtained (n = 40). The non-directed bronchoalveolar lavage and PLI measurements were performed within 3 hours postoperatively. A correlation with PLI was investigated in the samples with detectable uric acid levels (n = 16).

**Figure 7:**
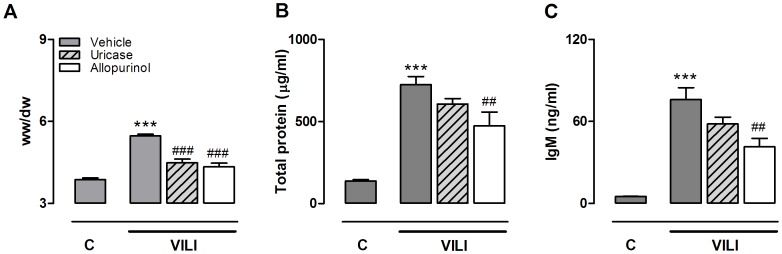
Allopurinol and uricase pre-treatment attenuate alveolar barrier dysfunction. Mice were pre-treated with vehicle (10% dimethylsulfoxide, dark grey bars), uricase (0.2 mg/kg, light grey, striped bars), or allopurinol (25 mg/kg (white bars) 1 hour before start of mechanical ventilation (VILI). Spontaneously breathing, vehicle pre-treated mice served as controls (C). After 5 hours mice were killed. Lung wet-to-dry ratio (**A**), total protein levels in lung lavage fluid (**B**) and immunoglobulin M (IgM) concentrations in (**C**) were analyzed. Data represent mean ± SEM of 4 control mice and n = 9 for the VILI groups (for IgM n = 5−6). ##P<0.01, ###P<0.001 vs. vehicle ventilated. ***p<0.001 vs. vehicle control.

**Figure 8:**
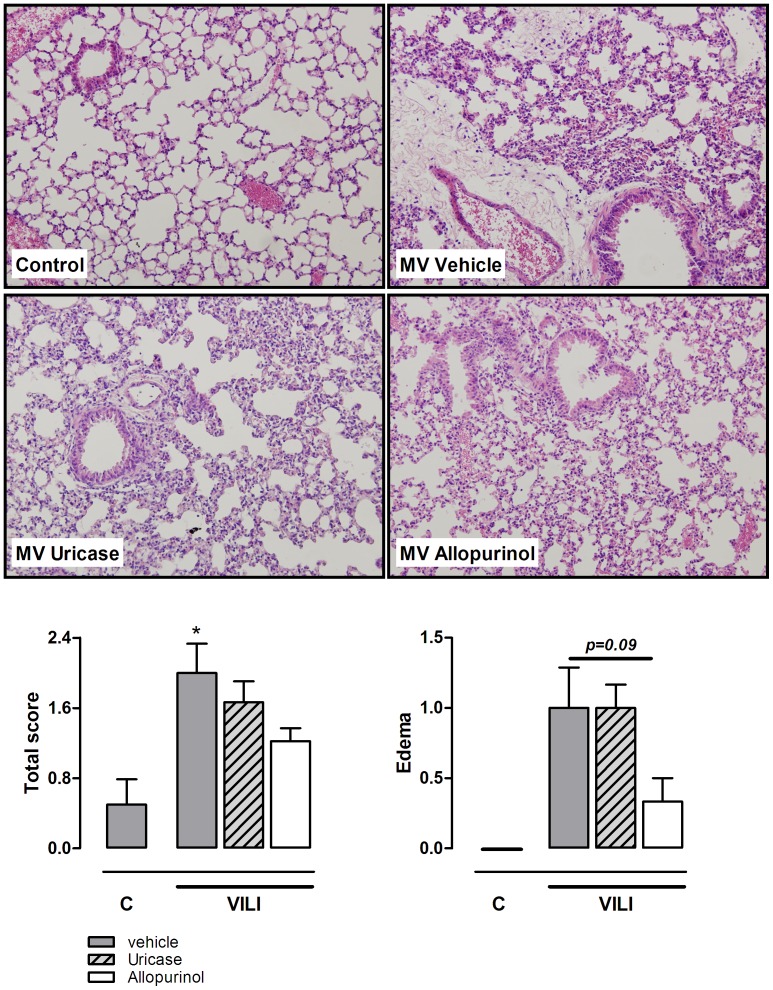
Histopathology of VILI in mice is affected by allopurinol pre-treatment. Lung tissue slides of mice pre-treated with vehicle (10% dimethylsulfoxide, dark grey bars), uricase (0.2 mg/kg, light grey, striped bars), or allopurinol (25 mg/kg (white bars) 1 hour before start of mechanical ventilation (VILI). Spontaneously breathing, vehicle pre-treated mice served as controls (C). Lungs were scored for presence of edema, hemorrhage, neutrophil influx, and hyaline membranes. Total scores and edema scores are demonstrated below. Data are presented as mean ± SEM of 4 control mice and n = 9 for the VILI groups. *p<0.05 vs vehicle control H&E staining, magnification ×20.

### Mice

#### Mice

The Animal Care and Use Committee of the Academic Medical Center, the Netherlands approved this study (permit number: 2010 LEICA102060-1). Animal procedures were carried out in compliance with Institutional Standards for Human Care and Use of Laboratory Animals. Male C57BL/6 mice aged 8–10 weeks were purchased from Charles River (Maastricht, The Netherlands). One hour before randomization to spontaneously breathing (n = 4/group) or MV (n = 9/group), mice received an intraperitoneal injection of allopurinol 25 mg/kg (Sigma-Aldrich, Schnelldorf, Germany) or uricase 0.2 mg/kg (Fasturtec, Sanofi Aventis, Notre Dame de Bondeville, France) or vehicle 10% dimethylsulfoxide/saline in 250 µl NaCl. Doses were based on a previous study demonstrating its efficacy in reducing bleomycin-induced lung inflammation in mice [13]. The experiment was performed twice: In the first subset, lungs were used for wet/dry ratio and to obtain BALF, in the second set of experiments lungs were used for tissue homogenate and lung histopathology. Methods used in this VILI model were published in detail previously [18].

#### Instrumentation, anesthesia, and monitoring

Mice received 1 hour before start of MV an intra peritoneal bolus of 1 ml normal saline. One hour thereafter, a Y–tube connector (1.0 mm outer diameter and 0.6 mm inner diameter, VBM Medizintechnik GmbH, Sulz am Neckar, Germany) was surgically inserted into the trachea under general anesthesia (intraperitoneal injection of 7.5 µl per 10 gram of body weight of: 1.26 ml 100 mg/ml ketamine, 0.2 ml 1 mg/ml medetomidine, and 1 ml 0.5 mg/ml atropine in 5 ml normal saline). During the 5 hours of MV maintenance anesthesia was given hourly (consisting of 10 µl per 10 gram body weight: 0.72 ml 100 mg/ml ketamine, 0.08 ml 1 mg/ml medetomidine and 0.3 ml 0.5 mg/ml atropine in 20 ml normal saline) via an intra peritoneal catheter (PE 10 tubing, BD, Breda, the Netherlands). Every 30 minutes 0.2 ml sodium bicarbonate (200 mmol/l NaHCO_3_) was administered via the same catheter to keep the mice hemodynamically stable and to prevent metabolic acidosis (18). Rectal temperature was maintained between 36.5–37.5°C using a heating pad. Blood pressure was monitored in the first subset of experiments at t = 0, t = 2.5 and t = 5 hours using a murine tail-cuff system. Data were recorded on a data acquisition system (PowerLab/4SP, ADInstruments, Spenbach, Germany).

#### Mechanical ventilation

Animals were placed in a supine position and connected to a mechanical ventilator (Servo 900 C, Siemens, Sweden). Simultaneously, 6 mice were pressure-controlled ventilated with settings known to induce VILI in mice (18): inspiratory pressure of 18 cm H_2_O (resulting in V_T_∼15 ml/kg), respiratory rate was 70 breaths/min, positive end-expiratory pressure was set at 2 cm H_2_O, the fraction of inspired oxygen was kept at 0.5 and inspiration to expiration ratio was set at 1∶1. At the end of the experiment mice were sacrificed by withdrawing blood from the carotid artery. Previous data from our lab using the same VILI model demonstrated adequate gas-exchange after 5 hours of MV [18]. Because of limited amounts, obtained plasma was stored for cytokine and chemokine measurements.

#### Sampling

BALF was harvested from the right lung by instilling 3 times 0.5 ml saline by a 22-gauge Abbocath-T cathether (Abbott, Sligo, Ireland). Cell counts were determined using a Coulter cell counter (Beckman Coulter, Fullerton, CA), differential cell counts were performed on cytospin preparations stained with Giemsa stain. Supernatant was stored at −20°C for further analysis. The left lung was weighed immediately after harvesting and dried for three days in a 65°C stove and weighed again for wet/dry ratio. In the second set of experiments right lungs were removed and snap frozen in liquid nitrogen. Next, these frozen specimens were homogenized in 4 volumes of saline and 50 µl was transferred in Tripure (Roche, Woerden, the Netherlands) for messenger ribonucleic acid (mRNA) analysis. The remaining homogenate was diluted 1∶1 in lysis buffer (150 mM NaCl, 15 mM Tris, 1 mM MgCl.H_2_O, 1 mM CaCl_2_, 1% Triton x-100, 100 µg/mL Pepstatin A, leupeptin and aprotinin, pH 7.4) and incubated at 4°C for 20 minutes. Cell free supernatants were obtained by centrifugation and stored at −80°C. Left lungs were fixed in 4% formalin and embedded in paraffin, 4 µm sections were stained with hematoxylin and eosin and scored by a pathologist who was blinded for group identity. To score lung injury 4 parameters were analyzed on a scale 0–4: alveolar congestion, hemorrhage, leukocyte infiltration, and thickness of the alveolar wall/hyaline membranes [18]. Score 0 represents normal lungs, 1, mild: <25% involvement, 2, moderate: 25–50% involvement, 3, severe: 50–75% involvement, and 4, very severe: >75% lung involvement. An overall VILI score was calculated based on the summation of all 4 scores.

#### Assays

Amplex Red Uric Acid Assay Kit (Molecular probes, Eugene, OR) with a detection limit of 0.43 µg/ml was used to analyze uric acid levels in samples. Total protein levels in BALF were determined using a Bradford Protein Assay Kit (OZ Biosciences, Marseille, France). Immunoglobulin M (IgM) measurements were performed as described previously [19], a detailed protocol is added to Data S4. Cytokines and chemokines were measured by enzyme-linked immunosorbent assay (R&D Systems Inc., Minneapolis, MN) according to manufacturers’ instructions. The detection limits were 8 pg/ml for KC, 31 pg/ml for IL-6, IL-1β, and tumor necrosis factor-α (TNF-α), and 47 pg/ml for macrophage inflammatory protein-2 (MIP-2).

#### mRNA expression analysis

Complementary deoxyribonucleic Acid (DNA) was synthesized with a reverse transcription reaction using oligo dT (Invitrogen) and Moloney murine leukemia virus reverse transcriptase (Invitrogen). Quantitative polymerase chain reactions were done using lightCycler®SYBR green I master mix (Roche, Mijdrecht, the Netherlands) and measured in a LightCycler 480 (Roche) apparatus using the following conditions: 5 min 95°C hot-start, followed by 40 cycles of amplification (95°C for 10 seconds, 60°C for 5 seconds, 72°C for 15 seconds), using primers for NLRP3, TLR2, TLR4, intracellular adhesion molecule-1 (ICAM-1), vascular adhesion molecule (VCAM), and e-selectin. For relative quantification we used the 2-ΔΔC_T_ method. Data were normalized for expression to the housekeeping gene hypoxanthine-guanine phosphoribosyl transferase (HPRT). For sequences see table 1.

#### Westernblotting

To study activation of intracellular innate immune pathways, we analyzed the degradation of inhibitory kappa B alpha (IκB-α), a parameter of nuclear factor-κB (NF-κB) activation. Lung tissue homogenate samples were measured for total protein concentration, and a total of 50 µg protein was separated by polyacrylamide gel electrophoresis (Criterion Bis-Tris 4–12% Precast Gel, Biorad, Veenendaal, the Netherlands). Proteins were transferred to a immobilon-P Transfer membrane (Millipore, Billerica, MA, USA), blocked in blocking buffer containing 5% non-fat dry milk proteins in TBS-T, washed, and incubated overnight with a rabbit polyclonal antibody against IκB-α (Santa Cruz Biotechnology, Cambridge, UK) at 4°C. The Ponceau protein staining was used as loading control. After washing, membranes were probed with peroxidase-labeled Rabbit-IgG1-HRP (Cell Signaling Technology, Danvers, MA) for 2 hours at room temperature in TBS. Again after washing, blots were imaged with Lumi-Light^Plus^ Western Blotting Substrate (Roche, Basel, Switzerland). Quantification was performed using ImageJ software (Rasband, W.S. USA), each band was corrected for total protein content using Ponceau concentrations. Protein levels did not differ between the experimental groups.

### Statistical Analysis

Statistical analyses were carried out using GraphPad Prism version 5 (Graphpad Software; San Diego, CA). Data are expressed as mean ± SEM unless stated otherwise. Two sample comparisons were performed by a Student-t test or Mann-Whitney U test, depending on data distribution. Categorical data were analyzed with the Chi Square test (Data S2 and S3), correlations were determined by Pearson’s analysis. To detect differences between multiple groups (non-ventilated controls versus ventilated vehicle pre-treated mice and ventilated vehicle pre-treated versus ventilated allopurinol or uricase pre-treated mice) ANOVA with Bonferroni post-hoc analysis was used. Non-parametric data were analyzed with a Kruskal-Wallis test, if overall significant, individual groups were assessed by Mann Whitney-U test. P values <0.05 were considered to be statistically significant.

## Results

### Uric Acid Levels in BALF of ALI Patients

Baseline characteristics of cardiac surgery patients developing transfusion-related ALI and their controls were described in detail previously [14] and are demonstrated in Data S2. Patients who developed ALI were older, received more transfusions, had a higher Euroscore and ASA score, and, total operation time, clamp-time and pump-time were longer when compared to controls. To investigate whether lung injury in patients is also associated with a rise in extracellular uric acid concentrations, we analyzed uric acid levels in lung lavage samples. Patients with lung injury had significantly higher uric acid levels in BALF when compared to patients without ALI (fig. 1A). In the second cohort of cardiac surgery patients in whom PLI measurements were performed, 1 patient met the criteria of (transfusion-related) ALI. Baseline characteristics are reported in Data S3. The mean PLI was elevated in all cardiac surgery patients [15]. In this cohort we also measured uric acid concentrations in BALF. Uric acid was elevated in 16 patients and below the limit of detection in 24 patients, which could not be explained by kidney function, ureum levels, or a medical history of smoking or alcohol abuses, all parameters that in theory could influence uric acid levels. When we analyzed the samples with measurable uric acid concentrations we found a positive correlation with the mean PLI levels (fig. 1B).

### Uric Acid Levels, TLR2, TLR4 and NLRP3 Gene Expression and IκB-α Protein Levels

All mice survived the duration of the experiment and heart rate and blood pressures remained stable without significant differences between the groups (fig. 2). In BALF, uric acid levels were significantly elevated by MV (fig. 3). Intra peritoneal uricase or allopurinol lowered uric acid levels. In plasma, concentrations were significantly reduced by allopurinol and uricase when compared to vehicle pre-treated animals (fig. 3). In BALF, uric acid concentrations were reduced by uricase. Uric acid levels in the allopurinol group were also lower but this did not reach statistical significance. Since previous work proposed that for optimal inflammation in response to uric acid crystals the receptors TLR2, TLR4, and the intracellular danger sensor NLRP3 are needed [13], we determined gene expression levels in lung tissue of these pattern recognition receptors. TLR2 and TLR4 mRNA levels were significantly elevated in lung tissue obtained from ventilated mice when compared to non-ventilated controls, NLRP3 mRNA levels demonstrated a non-significant increase (table 2). We did not detect differences in up-regulation of these DAMP signalling receptors between ventilated uricase or allopurinol pre-treated mice versus vehicle pre-treated mice (table 2). In addition, to analyze whether MV and/or treatment strategies influenced intracellular immune pathways we measured IκB-α protein levels in lung tissue homogenate as a proxy for NF-κB activation. Five hours of MV induced degradation of IκB-α as compared to non-ventilated controls (fig. 4). We observed no differences between ventilated treated and non-treated groups.

### No Effect of Uric Acid Inhibition upon MV-induced Inflammation

Five hours of injurious MV induced neutrophil migration to the alveolar compartment (fig. 5). Neutrophil influx was paralleled by up-regulated gene expression of the adhesion molecules ICAM-1, VCAM in lung tissue homogenate. Ventilated animals pre-treated with uricase or allopurinol demonstrated similar neutrophil counts in their BALF and up-regulation of the adhesion molecules were not attenuated as compared to vehicle pre-treated ventilated mice. To further dissect the pulmonary inflammatory response, cytokine and chemokine levels were measured in lung tissue homogenates and plasma (fig. 6). MV significantly enhanced IL-6, KC, and IL-1β levels in lung homogenates and IL-6 and KC levels in plasma. Concentrations of TNF-α and MIP-2 were not significantly affected by MV (data not shown). Uricase and allopurinol pre-treatment of ventilated mice did not attenuate IL-6, KC, or IL-1β levels in lung or plasma (fig. 6).

### Allopurinol and Uricase Pre-treatment Attenuate Lung Injury

In addition to the inflammatory component, alveolar barrier dysfunction is an important measure of VILI. Injurious MV induced lung edema as demonstrated by an increased pulmonary wet/dry ratio (fig. 7). We observed that uricase and allopurinol treatment both markedly reduced lung wet/dry ratio in ventilated animals. In line with these results, BALF total protein levels, significantly increased during ventilation, were attenuated in ventilated treated groups, reaching statistical significance for allopurinol pre-treated mice (fig. 7b). Alveolar-capillary permeability was further analyzed by measuring IgM levels in BALF. MV increased IgM levels as compared to non-ventilated controls. Allopurinol pre-treatment also significantly lowered IgM BALF levels compared to ventilated vehicle pre-treated mice (fig. 7c). Next, to study lung pathology, lung tissue slides were scored for signs for inflammation and damage (fig. 8). Five hours of MV induced mild histopathological changes compared to non-ventilated controls. None of the lungs showed signs of haemorrhage or hyaline membranes but edema and neutrophil influx were present. Total scores tended to be lower in allopurinol pre-treated animals. In addition, when focussing only on the edema scores, a clear trend (p = 0.09) towards reduced scores for allopurinol pre-treated animals was found.

## Discussion

Present findings demonstrate that uric acid levels are increased in BALF of patients with lung injury and that elevated uric acid concentrations are positively correlated with increased pulmonary vascular leakage in cardiac surgery patients. In an experimental VILI model we found that pre-treatment with allopurinol or uricase did not reduce pulmonary neutrophil influx, up-regulation of adhesion molecules and receptors involved in uric acid recognition, intracellular IκB-α degradation, or pulmonary and plasma cytokine levels. However, both treatment strategies attenuated lung wet/dry ratio. Allopurinol also reduced total protein and IgM levels in BALF and demonstrated a clear trend towards lower lung edema scores on histopathology, thus compatible with reduced lung injury.

In the early 90 s, urinary uric acid was measured as a marker for increased degradation of ATP and was found associated with the severity of human acute respiratory failure [20]. A more recent study analyzed uric acid levels in the alveolar compartment as a measure of antioxidant status in patients with ARDS [21]. Uric acid concentrations were found elevated, despite persistent oxidative stress, in ARDS patients compared to healthy controls. In line with these studies, we demonstrated increased BALF uric acid levels in cardiac surgery patients that developed ALI. These results cannot be directly compared to our animal experiments since in humans ALI was transfusion related and the circumstances in which ALI developed were different (for example: lungs were deflated and non-ventilated for several hours during the surgical procedure, and a cardiopulmonary bypass was used). However, we chose to present our results because it illustrates that human lung injury is also associated with a local rise in uric acid levels.

It has been known for decades that uric acid plays a central role in the painful inflammatory condition gout. The more recent discovery that released uric acid stimulates dendritic cell maturation and enhances the CD8+ T cell responses indicates its role in other forms of immune responses and inflammation [22]. Although the effects of soluble uric acid are still a matter of debate, it is well established that uric acid crystals strongly induce inflammation [23]. A study reported that TLR2, TLR4, and the NLRP3 inflammasome are needed for optimal sensing of uric acid crystals [13]. Activation of these pattern recognition receptors leads to increased release of pro-inflammatory mediators and neutrophil recruitment [13], [23]. Our results demonstrate that 5 hours of high tidal volume MV induced gene over-expression of these immune receptors, which is in line with previous reports [7], [8], [10]. These receptors can be activated by several endogenous ligands and it has been shown that besides uric acid other DAMPs such as hyaluronan, high mobility group box-1, and adenosine triphosphate are also increased during VILI [11]. Which DAMP contributes most is not established yet and the answer is probably dependent on tissue and type of injury. Modulation of uric acid levels with allopurinol or uricase during severe liver or lung injury substantially reduced inflammation, indicating that among the numerous potential contributing DAMPs uric acid was one of the more important ones in these experiments [13], [22]. This contrasts to our study where MV-induced inflammation was not affected by allopurinol or uricase treatment.

Uric acid has to be in the range of supersaturation (>70 ug/ml) to precipitate and form crystals [24]. All cells contain a high intracellular uric acid level as a result of the catabolism of purines. Concentrations further rise when injured cells degrade their DNA and RNA. The hypothesis is that in the close area of dying cells released uric acid exceeds its saturation point and crystallizes when it comes into contact with the high levels of free extracellular sodium. Preclinical studies have demonstrated that injurious MV can cause cell injury [3] and our data indeed show increased uric acid levels in BALF of ventilated mice. However, reported uric acid concentrations are low. These concentrations might be underestimated because the lavage procedure rigorously dilutes the liquid covering the alveolar epithelium and therefore local concentrations are unknown. However, it can also be speculated that in our relatively mild lung injury model released uric acid levels were not sufficient to induce crystals. Previous studies have demonstrated that lungs with pre-existing lung injury are more susceptible to the effects of MV. Future experiments might therefore focus on the potential of allopurinol and uricase in a 2-hit VILI model, in which an additional lung injurious stimulus, such as lipopolysaccharide or hydrochloride aspiration, is combined with MV. If the combined use of allopurinol and uricase has synergistic effects, since their mechanism of action is different but complementary, is another interesting question for further research.

Lung edema caused by increased alveolar epithelial and vascular endothelial permeability is an important hallmark of VILI and ARDS. Both allopurinol and uricase reduced lung wet/dry ratio, indicating that uric acid itself plays a role in MV-induced lung edema. It has been demonstrated that TLR4 and (NLRP3) inflammasome signalling mediate alveolar barrier dysfunction in VILI [9], [10], [25]. Triggering of the TLR pathway and NLRP3 inflammasome pathway (via IL-1β and IL-18) leads to intracellular IκB-α degradation and NF-κB activation. We observed no differences in IκB-α degradation between the ventilated treatment groups, indicating that the effect of allopurinol and uricase on lung edema was not NF-κB mediated. Uric acid may also function as pro-oxidant and generate free radicals in various reactions including the reaction with peroxynitrite [26]. Reactive oxygen and nitrogen species can modify or influence ion channels such as epithelial sodium channels, which are of utterly importance to maintain the pulmonary fluid balance [27]. It can be speculated that uric acid via the generation of free radicals had a cytotoxic effect on the endothelial-capillary membrane leading to an increased wet/dry ratio. In line with this hypothesis, we observed that elevated BALF uric acid levels correlate with higher levels of PLI. We did not study the source of the increased BALF uric acid levels. Since the mean PLI was elevated in all cardiac surgery patients, it can be postulated that the increased uric acid levels are not directly caused by the increased leakage itself.

The effect of allopurinol pre-treatment on barrier dysfunction was more pronounced indicating an additive role for xanthine oxidoreductase. Allopurinol blocks uric acid synthesis by inhibiting the enzyme xanthine oxidoreductase. This enzyme catalyzes the 2 terminal steps of purine degradation (hypoxanthine -> xanthine -> uric acid) and has also the ability to generate reactive oxygen species. Our allopurinol data are in line with a previous experimental study where 2 hours of injurious MV (20 ml/kg) resulted in increased pulmonary xanthine oxidoreductase activity [28]. Allopurinol pre-treatment of these mice attenuated BALF protein concentrations and evans blue dye extravasation in the alveolar compartment. In a different study this group reported that allopurinol also attenuated MV-induced alveolar cell apoptosis [29]. It is thought that its role in MV-induced capillary leakage is related to the production of reactive oxygen species that react with nitric oxide to form ONOO^-^, a powerful oxidant [28]. Whether reactive oxygen species indeed play an important role in the mechanisms via which allopurinol improves barrier dysfunction should be a focus of future research.

Our study has limitations. First, modulating uric acid concentrations with allopurinol or uricase has limitations. Blocking xanthine oxidoreductase by allopurinol only partially reduces uric acid concentrations as levels are also affected by absorption from diet [12]. Uricase, an enzyme that oxidizes uric acid into allantoin and water, has pharmacokinetic limitations since it is short lived and only slowly distributed to interstitial fluids [12]. It is therefore possible that uric acid’s contribution is underestimated in our model. However, in a bleomycin-induced lung injury model both allopurinol and uricase treatment, using the same dosages, did reduce acute lung inflammation [13]. Second, a direct comparison between the mouse and the human studies is not possible because multiple factors may have influenced ALI development in patients. In contrast, we used healthy animals in our murine model and lung injury was solely induced by injurious ventilation. Third, in our murine model mice received allopurinol or uricase before lung injury was established and therefore we did not study if allopurinol and uricase also have therapeutic effects. Fourth, the tidal volumes used in our VILI model are higher then those normally used in clinical practice. Indeed, to date the only clinically proven strategy to reduce morbidity and mortality in ARDS patients is low tidal volume MV [4]. However, recent computed tomography studies demonstrated in ARDS that the distribution of pulmonary aeration and inflammation is very heterogenous [30], [31]. Even low tidal volume MV can cause overdistention in the aerated compartments [31]. VILI is therefore considered a regional phenomenon and experimental models using high tidal volume MV still reveal important information on VILI pathogenesis.

In conclusion, we found that allopurinol and uricase pre-treatment were not capable of reducing innate immune activation and inflammation associated with MV in our murine VILI model. However, uric acid seems to play an important role in the pathogenesis of MV-induced lung edema. Future experimental studies are needed to elucidate the underlying mechanisms.

## Supporting Information

Data S1 **methods human studies.**
(DOC)

Data S2 **ALI versus no ALI cohort.**
(DOC)

Data S3 **PLI cohort.**
(DOC)

Data S4 **IgM ELISA.**
(DOC)
