# Supplementary material for: Pre-Treatment with Allopurinol or Uricase Attenuates Barrier Dysfunction but Not Inflammation during Murine Ventilator-Induced Lung Injury
Source: PLoS One. 2012 Nov 30;7(11):e50559. doi: 10.1371/journal.pone.0050559 (PMC3511544; doi:10.1371/journal.pone.0050559)
Supplement: Data S1 — methods human studies. (DOC) [file pone.0050559.s001.doc]

**Supplemental digital information**

**Supplemental data S1: methods human studies**

*Cardiothoracic surgery/anesthesia*

The studies were performed in a Dutch university hospital (1;2). Patients were anesthetized according to institutional protocol: lorazepam was used as premedication followed by etomidate, sufentanil, and rocuronium for induction of anesthesia and facilitation of the intubation procedure. During surgery, sufentanil was used as analgesic and sevoflurane plus propofol were used as maintenance anesthesia. Muscle relaxants were not used during the surgical procedure. At the end of the procedure small doses of morphine and midazolam could be given. Steroids were used at the discretion of the cardio-anesthesiologist. The cardiopulmonary bypass was performed under mild to moderate hypothermia (28-34°C) using a membrane oxygenator and a non-pulsatile blood flow. Lungs were deflated during the procedure. After surgery, patients were transferred to the intensive care unit (ICU) with mechanical ventilation.

*ICU management*

The ICU protocol included fluid infusion with normal saline and starch solutions, blood transfusions to maintain hemoglobulin concentrations (≧5 mmol/l), dopamine and norepinefrine in continuous infusion to keep the mean arterial blood pressure ≧ 65 mm Hg, and dobutamine and/or milrinone to achieve a cardiac index ≧ 2.5 l/min/m^2^ or a mixed venous oxygenation > 60%. Propofol was infused until core temperature was 36.0° C. Acetaminophen and morfine were used as analgesics.

Acute lung injury (ALI) was defined by a new onset of hypoxemia or deterioration demonstrated by a PaO_2_/FiO_2_ < 300 mmHg, with bilateral pulmonary changes on chest X-ray, in the absence of elevated left arterial pressure defined as a pulmonary occlusion pressure < 18 mmHg. Chest radiographs were taken before surgery and on arrival at the ICU. Two independent physicians scored these radiographs for the presence of new onset bilateral interstitial abnormalities. When the interpretation was different, the chest radiograph and the description by the radiologist were reviewed to obtain consensus. ALI was considered transfusion related when it developed within 6 hours after transfusion. At onset of ALI, a non-directed bronchoalveolar lavage was performed, controls were lavaged within 30 hours after ICU admission. Via the orotracheal tube, a 50 cm 14 Fr tracheal suction catheter was inserted (3). The tube was advanced until resistance was encountered, then 20 ml 0.9% saline was instilled over 10 seconds and immediately aspirated. Bronchoalveolar lavage fluid was centrifuged and supernatant was stored at -80° C until further analysis. Patients included in the study were observed for the onset of ALI up to 30 hours after ICU admission.

References

(1) Vlaar AP, Hofstra JJ, Determann RM, Veelo DP, Paulus F et al. (2011) The incidence, risk factors, and outcome of transfusion-related acute lung injury in a cohort of cardiac surgery patients: a prospective nested case-control study. Blood 117:4218-4225.

(2) Vlaar AP, Cornet AD, Hofstra JJ, Porcelijn L, Beishuizen A et al. (2012) The effect of blood transfusion on pulmonary permeability in cardiac surgery patients: a prospective multicenter cohort study. Transfusion 52:82-90.

(3) Schultz MJ, Millo J, Levi M, Hack CE, Weverling GJ et al. (2004) Local activation of coagulation and inhibition of fibrinolysis in the lung during ventilator associated pneumonia. Thorax 59:130-135.
