# Supplementary material for: Pre-Treatment with Allopurinol or Uricase Attenuates Barrier Dysfunction but Not Inflammation during Murine Ventilator-Induced Lung Injury
Source: PLoS One. 2012 Nov 30;7(11):e50559. doi: 10.1371/journal.pone.0050559 (PMC3511544; doi:10.1371/journal.pone.0050559)
Supplement: Data S2 — ALI versus no ALI cohort. (DOC) [file pone.0050559.s002.doc]

**Supplemental data S2: ALI versus no ALI cohort**

A total of 1000 cardiac surgery patients were screened for inclusion from November 2006 until February 2009, of these, 668 patients were included in the study. Sixteen patients developed ALI, all within 6 hours of transfusion. Controls (n=62) were randomly matched with cardiac surgery patients not developing ALI.

**Table: Patient characteristics ALI vs No ALI cohort**

|  | **No ALI**  **(n=62)** | **ALI**  **(n=16)** |
| --- | --- | --- |
| **Pre-operative** |  |  |
| Age # | 68 (61-74) | 74 (70-80)** |
| Male gender, n (%) | 47 (76) | 12 (75) |
| Euroscore # | 4.5 (4.3-9.8) | 6.0 (3.0-6.0)* |
| ASA # | 3.0 (3.0-3.0) | 3.0 (3.0-3.8)* |
| FEV1 # | 90 (76-106) | 82 (61-101) |
| Left ventricular function: |  |  |
| Poor, n (%) | 6 (10) | 1 (6) |
| Moderate, n (%) | 11 (18) | 7 (44)* |
| Good, n (%) | 43 (69) | 9 (50) |
| Alcohol abuse, n (%) | 2 (3) | 1 (6) |
| Smoking, n (%) | 18 (29) | 2 (13) |
| Ureum mmol/l # | 6 (5.0-8.0) | 6.5 (5.3-9.8) |
| Creatinine mmol/l # | 83 (73-108) | 88 (73-92) |
| **Surgery** |  |  |
| CABG, n (%) | 33 (53) | 6 (38) |
| Valve replacement n (%) | 24 (39) | 9 (56) |
| Other type of surgery n (%) | 5 (8) | 1 (6) |
| Clamp time, min † | 79 (34) | 115 (42)*** |
| Pump time, min # | 115 (89-139) | 170 (126-198)*** |
| Operation time, min # | 244 (205-294) | 359 (273-416)*** |
| Amount of transfusions, n # | 1 (0-4) | 6 (3-10)*** |
| **Outcome** |  |  |
| ICU LOS (hrs) # | 42 (24-52) | 102 (70-172)*** |
| Mechanical ventilation (hrs) # | 17 (11-24) | 25 (21-83)*** |

Acute lung injury (ALI); EuroSCORE: European System for Cardiac Operative Risk Evaluation; ASA-score: physical status classification system according to the American Society of Anesthesiologists; FEV1: forced expiratory volume in 1 second, given in % of predicted, data are presented in † mean (SD) or in # median (IQR) when appropriate. *p<0.05, **p<0.01, ***p<0.001
