# Supplementary material for: Pre-Treatment with Allopurinol or Uricase Attenuates Barrier Dysfunction but Not Inflammation during Murine Ventilator-Induced Lung Injury
Source: PLoS One. 2012 Nov 30;7(11):e50559. doi: 10.1371/journal.pone.0050559 (PMC3511544; doi:10.1371/journal.pone.0050559)
Supplement: Data S3 — PLI cohort. (DOC) [file pone.0050559.s003.doc]

**Supplemental data S3: PLI cohort**

Sixty patients were included in the original study which was performed in 2 university hospitals in the Netherlands. In this study the effect of transfusion on pulmonary leakage index in cardiac surgery patients was investigated. For the present cohort we included patients in which a PLI measurement and a bronchoalveolar lavage procedure were performed (n=40).

**Table: Patient characteristics PLI cohort**

|  | **uric acid**  **(n=16)** | **no uric acid**  **(n=24)** |
| --- | --- | --- |
| **Pre-operative** |  |  |
| Age † | 64 (12) | 68 (11) |
| Male gender, n (%) | 11 (71) | 17 (69) |
| Euroscore # | 4 (3-6) | 5 (3-6) |
| ASA # | 3.0 (3.0-3.0) | 3.0 (3.0-3.0) |
| FEV1 † | 91 (16) | 99 (18) |
| Left ventricular function: |  |  |
| Poor, n (%) | 1 (6.3) | 0 (0) |
| Moderate, n (%) | 4 (25) | 7 (29) |
| Good, n (%) | 8 (50) | 16 (67) |
| Alcohol abuse, n (%) | 0 (0) | 0 (0) |
| Smoking, n (%) | 3 (19) | 5 (21) |
| Ureum mmol/l # | 5.0 (5-7) | 6.5 (6-9) |
| Creatinine mmol/l # | 75 (69-88) | 85 (75-97) |
| **Surgery** |  |  |
| CABG, n (%) | 8 (50) | 16 (67) |
| Valve replacement n (%) | 6 (38) | 5 (21) |
| Other type of surgery n (%) | 1 (6) | 2 (8) |
| Clamp time, min † | 76 (34) | 75 (35) |
| Pump time, min † | 112 (50) | 107 (38) |
| Operation time, min # | 239 (213-358) | 246 (194-291) |
| Amount of transfusions, n # | 1.5 (0-2) | 1 (0-5.8) |
| **Outcome** |  |  |
| PLI | 32.1 (17) | 30.6 (18) |
| ICU LOS (hrs) # | 45 (24-54) | 42.5 (25-50) |
| Mechanical ventilation (hrs) # | 14 (7-22) | 14 (10-18) |

Acute lung injury (ALI); EuroSCORE: European System for Cardiac Operative Risk Evaluation; ASA-score: physical status classification system according to the American Society of Anesthesiologists; FEV1: forced expiratory volume in 1 second, given in % of predicted, data are presented in † mean (SD) or in # median (IQR) when appropriate. *p<0.05, **p<0.01, ***p<0.001
