# Supplementary material for: Pre-Treatment with Allopurinol or Uricase Attenuates Barrier Dysfunction but Not Inflammation during Murine Ventilator-Induced Lung Injury
Source: PLoS One. 2012 Nov 30;7(11):e50559. doi: 10.1371/journal.pone.0050559 (PMC3511544; doi:10.1371/journal.pone.0050559)
Supplement: Data S4 — IgM ELISA. (DOC) [file pone.0050559.s004.doc]

**Supplemental data S4: IgM ELISA**

Immunoglobulin M (IgM) was measured using 96 wells plates coated with Anti-mouse Ig (SouthernBioTech, Birmingham, AL, USA) in sodium carbonate buffer at 4 °C overnight (1). Plates were washed and blocked with 5% bovine serum albumin (BSA, Roche Applied Science, Indianapolis, IN, USA) in PBS for 1 hour at room temperature. After washing, the plates were incubated with diluted samples in 1% BSA and 0.05% Tween 20 (Sigma-Aldrich) in PBS for 2 hours in 37 °C. For detection we used anti-mouse IgM-HRP (SouthernBioTech, Birmingham, AL, USA) and plates were developed with 3,3′,5,5′-tetramethylbenzidine (TMB; Invitrogen, Paisley, UK) and 0.003% H_2_O_2_. Optical density of each well was measured at 450 nm.

Reference

(1) Reijmers RM, Groen RW, Kuil A, Weijer K, Kimberley FC et al. (2011) Disruption of heparan sulfate proteoglycan conformation perturbs B-cell maturation and APRIL-mediated plasma cell survival. Blood 117:6162-6171.
